# Reducing craving and lapse risk in alcohol and stimulants dependence using mobile app involving ecological momentary assessment and self-guided psychological interventions: Protocol for a randomized controlled trial

**DOI:** 10.3389/fpsyt.2022.1011585

**Published:** 2022-12-14

**Authors:** Katarzyna Obarska, Alicja A. Binkowska, Przemysław Marcowski, Karol Szymczak, Karol Lewczuk, Katarzyna Sollich, Maria Banaszak, Bohdan Woronowicz, Małgorzata Nowicka, Maciej Skorko, Mateusz Gola

**Affiliations:** ^1^PredictWatch, Białystok, Poland; ^2^Institute of Psychology, Polish Academy of Sciences, Warsaw, Poland; ^3^DrugsTeam, NeuroCognitive Research Center, SWPS University of Social Sciences and Humanities, Warsaw, Poland; ^4^Institute of Psychology, The Maria Grzegorzewska University, Warsaw, Poland; ^5^Institute of Psychology, Cardinal Stefan Wyszynski University in Warsaw, Warsaw, Poland; ^6^Monar Association, Warsaw, Poland; ^7^Consulting Center Akmed, Warsaw, Poland

**Keywords:** mHealth, addiction, cognitive-behavioral therapy, mobile phone apps, EMA, substance use disorder (SUD), psychological intervention, mindfulness

## Abstract

**Background:**

The prevalence of alcohol consumption in Poland is estimated to be as high as 80% of the adult population. The use of stimulants is the second most common reason for seeking addiction treatment. However, treatment outcomes remain unsatisfactory, as 40–85% of individuals who complete various treatment programs relapse and fall back into addiction within 2 years following program completion.

**Methods:**

The 13-armed randomized controlled trial aimed to assess the effectiveness of a mobile app-based self-guided psychological intervention delivered *via* a smartphone app (*Nałogometr*) in reducing craving and lapse risk in problematic alcohol or stimulants use. Participant recruitment and data collection will be performed from June 2022 to September 2022. The 4-week mobile intervention program will include short-term and long-term intervention modules based mainly on mindfulness and cognitive-behavioral therapy. Intervention effectiveness assessment will include Ecological Momentary Assessment. That is, we will collect longitudinal data on a set of characteristics of day-to-day functioning. The primary outcomes will include a self-reported number of lapses and addiction craving level. In contrast, the secondary outcomes will be the severity of problematic substance use, anxiety and depression scores, and life satisfaction scores.

**Conclusion:**

This study will establish how mobile app-based self-guided psychological interventions can help reduce craving and lapse risk in alcohol and stimulant dependence. If successful, this randomized controlled trial (RCT) may provide an innovative, easily available, and cost-effective mHealth approach for craving and lapse risk in substance addictions.

**Clinical trial registration:**

[https://clinicaltrials.gov/], identifier [NCT054 34429].

## 1 Introduction

Addiction is a highly prevalent and chronic disorder, harming individuals and society by placing a significant burden on social policy and medical resources. A major issue in addiction is its relapsing nature, with the propensity to relapse long after the more obvious signs of acute withdrawal have abated (1). Alcohol and stimulants (including amphetamines, methamphetamine, or methcathinone) dependencies manifest by an impaired ability to control use, increasing priority over other activities, and persistence of use despite harm or negative consequences, often accompanied by a craving to use substance (2). The physiological symptoms of addiction include tolerance, withdrawal symptoms, or repeated use of substances to prevent withdrawal symptoms. Addiction is usually diagnosed if alcohol or substance use occurs continuously for at least 3 months or if dependence symptoms occur for at least 12 months.

Globally, in the adult population (aged ≥ 15 years), a person consumes 6.43 liters of pure alcohol per year (3). Approximately 18.4% of the adult population reported heavy episodic drinking (≥ 60 g of alcohol on one occasion) in the past 30 days. Central, Eastern, and Western Europe reported higher alcohol consumption and a higher percentage of heavy consumption among users (49.5, 46.9, and 40.2%, respectively) than North Africa and the Middle East. Central sub-Saharan Africa showed the highest proportion of heavy consumption (78.9%) with a relatively low per capita consumption (3). Alcohol addiction was the most prevalent substance of dependence, with 63.5 million cases in 2015. The highest age-standardized rate of alcohol dependence was in Eastern Europe (2,786.7 per 100,000 people) and the lowest in North Africa and the Middle East (274.2 per 100,000 people). In 2018, the rate of alcohol use in Poland was 79.9%, while the percentage of drug use was 5.4% (population aged 15–64). The highest prevalence of drug use was registered in Spain (11.9% in 2017), Netherlands (11.8% in 2018), and France (11.4% in 2017). The lowest prevalence of alcohol use was in Cyprus (2.2% in 2016) and Hungary (2.3% in 2015).

The global prevalence of amphetamines and cocaine use is about 0.77 and 0.35%, respectively (4). In 2015, amphetamine and cocaine dependence was estimated to be 6.6 million and 3.9 million cases. The region of North America had one of the most prevalent rates of cocaine dependence, 301.2 per 100,000 people. Australia and New Zealand had the highest prevalence of age-standardized rates of amphetamine dependence (491.5 per 100,000 people) and high rates of cocaine use dependence (160.6 per 100,000 people). Age-standardized amphetamine and cocaine dependence prevalence were lowest in central sub-Saharan Africa and eastern and western sub-Saharan Africa. In the population aged 15–34, the prevalence of drug use in the last year was higher than in the entire population (aged 15–64). The highest rates were in France (22.6% in 2017), Netherlands (21.5% in 2018), and Italy (21.3% in 2017), and the lowest in Hungary (3.5% in 2015), Cyprus (4.3% in 2016) and Greece (4.5% in 2015) (5). In Poland, the proportion of drug users aged 15–34 was 10.4% (2018). Among the general population, 4% of respondents (15–34 years old) admitted to using amphetamines or cocaine (0.5%) in the last year, with a higher rate among men than women. Amphetamines are the second drug (after cannabis), because of which people started treatment in Poland (27%), followed by methamphetamine (9%) and cocaine (2.2%) (6).

Maintaining abstinence is one of the primary goals of addiction treatment. However, treatment outcomes remain unsatisfactory, primarily due to the high rate of relapses. It is estimated that 40–80% of individuals who successfully complete various treatment programs relapse and fall back into addiction (7, 8). Among different approaches, cognitive-behavioral therapy (CBT) and cue-exposure therapy (CET) have proven to be effective in treating substance additions (9). CBT can help cope with mood disturbances and addiction cravings by restructuralization of maladaptive beliefs and behaviors related to addiction and can also be effective in treating substance use disorders in clinical settings in combination with pharmacotherapy (10). The additional option is a computer-assisted CBT, as computers and mobile devices with Internet access are now readily available. A six-module computer-assisted CBT program effectively adjuncts to standard substance use disorder therapy (11). The most significant advantage of such a solution is that it is much cheaper than standard CBT and does not require constant access to clinicians.

Mindfulness integrates well with dialectical behavior therapy (DBT) and acceptance and commitment therapy (ACT) which can be adapted for treating substance and behavioral addictions (12, 13). These approaches in psychotherapy focus on acceptance, cognitive defusion, and flexible attention to the present (14). Standardized mindfulness techniques effectively reduce emotional distress and alleviate symptoms of psychiatric disorders (15). Mindfulness-Based Interventions (MBIs) are used in treating substance use disorders (nicotine, alcohol, cocaine, and opioids) as they address the mechanisms of addiction. MBIs programs usually last 8 weeks and target mindfulness skills to deal with addiction in different situations of everyday life, e.g., by being mindful of one’s craving (16). Mindfulness techniques such as focused attention or open monitoring aim to train attentional reorienting skills, metacognition, reappraisal, and inhibitory control, which are essential in coping with addictive behaviors (17). Mindfulness meditation reduces psychological distress, decreases rumination (18), negative affect and state anxiety, and increases positive affect (19). MBIs were found effective regarding withdrawal symptoms, craving, and negative consequences of substance use (20). Although Mindfulness-based Relapse Prevention is gaining recognition as a therapeutic strategy, yielding minor effects on withdrawal or craving versus comparator interventions, further data are needed to confirm it as an effective strategy. CBT, MBIs, and treatment as usual (TAU) did not differ in substance use frequency and relapse. ACT effectively reduces substance use (21), and the effects of MBIs and ACT on alcohol dependence did not differ from other standardized treatment approaches.

Journaling is a common therapeutic tool used in two forms—expressive writing and gratitude journaling (22). The first journaling technique is based on writing about crucial thoughts and emotions for about 20 minutes in up to 4 sessions (23). Gratitude journaling is a diary that includes everything a patient is grateful for and aims to focus on the positive aspects of everyday life (24). The low risk of adverse effects, low resource requirement, and focus on self-efficacy make journaling a tool that can be easy to combine with other evidence-based forms of therapy (25). There needs to be evidence-based guidance about the utility of journaling as a non-pharmacological treatment. There are only limited efforts of a systematic review of the efficacy of journaling (25). We should note that there are studies indicating no benefits from journaling when using alcohol or illegal drugs (23), and there are concerns that journaling is associated with becoming a passive observer, too conscious, or overthinking. Self-monitoring involves being aware of emotions and behaviors in response to different situations, and it is the ability to regulate and modify behaviors in response to various factors. Internet-based self-monitoring interventions offer high usability, accessibility, interactive multimedia, graphical features, tracking systems, sensor-based devices, and individualized feedback (26). Digital diaries for treating bipolar disorder, pain, weight and sleep management, chemotherapy, and borderline personality disorder were promising and usable in digital self-monitoring (26). Mobile phone applications combined with new possibilities of self-monitoring increase data quality, enhance participants to registration, examine the risk and protective factors during therapy, and investigate the predictors of successful treatment.

Ecological Momentary Assessment (EMA) involves the repeated sampling of subjects’ current behaviors and experiences in real time in natural environments. EMA holds the unique promise to advance the science and practice of clinical psychology. Surveys were a traditional tool in research on addictions, but they are only point measurements that do not capture the dynamics of changes in behavior. EMA was used in tobacco and alcohol use disorders and relatively less often in heroin and cocaine research regarding both in-treatment and recovery patients (27). In methamphetamine- or cocaine-dependent participants, studies with EMA assessed stress and the cognitive, affective, and motivational factors on drug craving (28–30). EMA used in self-monitoring was effective and reliable in treating and assessing eating disorder urges (31). EMA and self-monitoring methods support behavior change and self-management by increasing the awareness of addictive behaviors and triggers, goal progress tracking, self-rewards, reminders, reinforcement, and personal feedback (32).

Due to the high risk of relapse in addiction (33), mobile health (mHealth) interventions could be a valuable tool for enhancing the post-therapeutic effects after treatment (34). The mHealth interventions delivered through smartphone apps are gaining popularity; however, the evidence for their effectiveness often remains unsolved (35). With over 300,0000 health applications available (36), a small number have been clinically tested before entering the market (37). Currently, there are no evidence-based mobile apps in Poland that effectively reduce craving and lapse risk in substance use disorders. The present study aims to test the effectiveness of mobile app-based self-guided psychological intervention delivered *via* a smartphone app (*Nałogometr*) involving EMA in reducing craving, lapse risk, and substance use (alcohol, stimulants).

## 2 Methods

### 2.1 Aim

The study aimed to evaluate the effectiveness of long-term and short-term intervention modules in reducing craving and lapse levels in alcohol and stimulant problematic use.

### 2.2 Participants

Recruitment takes place from June 2022 until September 2022 to ensure the targeted sample size is reached. Participants will be recruited *via* newsletters, advertisements, and social media. The study will be conducted using the proprietary mobile application *Nałogometr* to collect all relevant measurements. Inclusion criteria will include self-reported problematic alcohol or stimulant use, being 18 or over, speaking Polish fluently, using Android or iOS smartphones, and accepting informed consent. We recruit only people living in Poland.

### 2.3 Sample size calculation

To determine the sample size, a simulation-based power analysis was performed. For the simulation, we assumed a linear mixed-effects model (with random participant-level intercepts), 21 data points (EMA entries) per participant. Based on the results of previous substance use reduction mobile intervention studies [for a review, see Staiger et al. (38)], small effect size of the treatment, i.e., introducing an intervention, was also assumed in the simulation. This analysis suggested that a minimum sample size of 650 participants will be necessary to achieve 80% power of detecting a treatment effect. Due to the nature of the study, i.e., a nationwide study of problematic substance (alcohol and stimulants) use, our sampling strategy has been designed. to maximize the demographic diversity of the resulting sample, particularly with respect to demographic characteristics (e.g., gender, age, geographic and socioeconomic setting) within each addiction profile.

### 2.4 Study design

The study was pre-registered within the Open Science Framework (OSF) repository.^1^

### 2.5 Procedure

The study will be conducted *via* the mobile application *Nałogometr*, a proprietary custom-design mobile app available from Google Play or the App Store. The download and onboarding process in the app will be used as baseline data in the study. Upon first use, the app will automatically navigate the participant through necessary permissions and consent about the collection of different types of data. After logging in, participants will be asked to complete a baseline onboarding questionnaire that collects demographic information and their substance use or behavioral habits.

### 2.6 Randomization

Randomization will be applied using an automated balancing algorithm. The algorithm will aim to partition participants into groups based on the initial onboarding assessment to make the resulting groups as balanced as possible concerning the characteristics outlined in the study design section, i.e., (1) primary addiction type; (2) participation in addiction-related therapy; (3) gender; (4) age; (5) addiction severity; (6) abstinence duration. Following randomization, participants in groups 1–12 will receive access to the intervention modules following a 5-day delay. In conditions 1:10, participants get access to the short-term intervention module and one type of long-term intervention. In condition 11, participants get access to the short-term intervention module and all long-term interventions. In condition 12, participants get access only to the short-term intervention module.

**FIGURE 1:**
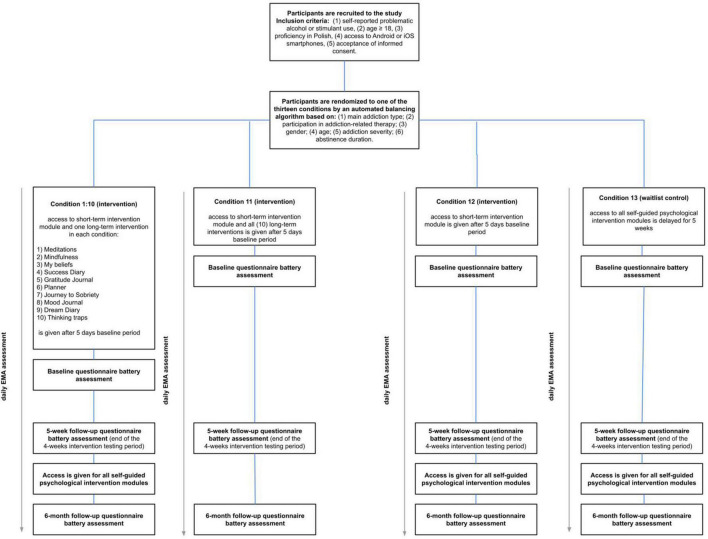
Participant flow chart.

Participants in the waitlist control group will be able to access all intervention materials 5 weeks following study enrollment (see Figure 1).

The study will take place from June 2022 until September 2022, with ongoing participant recruitment. The study includes ecological momentary assessment (EMA), assessing several variables important linked to lapse risk. Participants will be asked three times daily to answer questions about their mood, psychological and physiological state, craving, and if lapses occur. EMA will be administered daily between 6 a.m. and 12 a.m (morning EMA) and between 4 p.m. and 10 p.m. (evening EMA). Participants can select a specific time for assessment delivery that best suits their schedule and needs. When prompted to complete the EMA, participants will have the time to complete it until the next scheduled. Assessments are designed to take less than 2 min to complete. In addition, participants will be able to initiate assessments themselves to not skip any measurements for the day.

### 2.7 Digital therapeutic app *Nałogometr*

#### 2.7.1 Mobile application

*Nałogometr*^2^ is a mobile application dedicated to reducing craving and lapse risk among users with substance use disorder or problematic substance use. The Predictwatch company creates the app. The app includes a dashboard, sobriety calendar, EMA modules, and self-guided psychological interventions. A team of scientists and addiction treatment specialists created the content.

##### 2.7.1.1 Dashboard

The mobile app dashboard is simple and easy to use, allowing quick access to EMA, sobriety calendar, and information about sober and not-sober days. Access to self-guided psychological interventions and weekly feedback is also available.

##### 2.7.1.2 Weekly feedback reports

The mobile app automatically processes and analyzes EMA data entered by the user and generates personalized feedback for the past 7 days. Reports contain information about sober and non-sober days, changes in craving and lapse risk relative to the previous week, and finally, the relationships between craving and the three most important EMA items and protective factors.

##### 2.7.1.3 Self-guided psychological intervention modules

**FIGURE 2:**
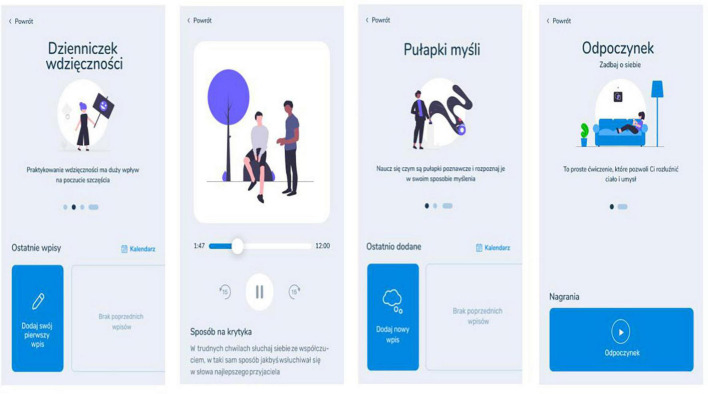
Example screenshots of the self-guided intervention modules in *Nałogometr*.

Two main self-guided intervention modules were created for a mobile app: (1) short-term self-guided intervention module and (2) long-term self-guided intervention module (Table 1 and Figure 2). The delivery of these interventions will be initiated 5 days following study enrollment.

**TABLE 1:** Intervention modules in the mobile app.

Intervention modules	Description
Short-term interventions	This module consists of audio-guided sessions on gratitude, auto-empathy, thoughts management, and relaxation. Moreover, they are based on breath relaxation exercises, craving management, and motivation to change.
**Long-term interventions**	
Meditations	Audio-guided meditation sessions focused on increasing the awareness of emotions, effectively reading body signals, and coping with stress.
Mindfulness	Audio-guided sessions on mindfulness of the breath, body, thoughts, and emotions.
My beliefs	Thought management technique based on CBT
Thinking traps	Thought management technique based on CBT (reframing thoughts)
Journey to sobriety	Audio-guided sessions
Planner	Improves goal achievement and self-efficacy.
Mood journal	Improves understanding of the relationship between situations, thoughts, mood and sobriety.
Dream diary	Improves self-observation and awareness of emotions.
Success diary	Enhances self-confidence and self-esteem.
Gratitude journal	Enhances positive attitude.

### 2.8 Measures

Sociodemographic data include gender, age, and place of residence. Substance use-related questions include frequency of use, lifetime use, treatment seeking, and abstinence period (see Supplementary Tables 1, 2). The study includes EMA (three times a day) assessing several variables: craving, lapses, mood, arousal, pressure, anxiety, procrastination, loneliness, tiredness, anger, hunger, and uncertainty (see Supplementary Table 3).

Moreover, the questionnaire assessment will occur three times during the study period (see Table 2).

**TABLE 2:** Timeline of the questionnaire assessment.

Assessment instrument	Baseline (week 1)	Follow-up (week 5)	Follow-up (month 6)
Socio-demographic questions	*x*		
Substances use-related measures			
Psychological dependence (SDS)	*x*	*x*	*x*
Alcohol (AUDIT)	*x*	*x*	*x*
Stimulants (DUDIT)	*x*	*x*	*x*
Psychopathological symptoms measures			
Depression and anxiety (HADS)	*x*	*x*	*x*
Psychological functioning measures			
Sensation seeking (BSSS)	*x*	*x*	*x*
Emotion regulation (DERS)	*x*	*x*	*x*
Impulsivity (UPPS-P)	*x*	*x*	*x*
Coping with stress (Mini-COPE)	*x*	*x*	*x*
Life satisfaction (SWLS)	*x*	*x*	*x*

#### 2.8.1 Primary outcome

The primary outcomes of interest are the self-reported lapses (number of lapses reported in daily EMA) and addiction craving (intensity of the urge to use a given substance at the moment of completing the assessment) (see Supplementary Table 3).

#### 2.8.2 Secondary outcomes

Problematic alcohol use is measured with an Alcohol Use Disorders Identification Test (AUDIT) (39), a 10-item one-dimensional tool. Participants answer the questions in terms of standard drinks units. AUDIT assesses the amount and frequency of alcohol intake (items 1–3), alcohol dependence (questions 4–6), and problems related to alcohol consumption (items 7–10). Questions 1 to 8 are scored on a 5-point scale ranging from 0 to 4, and questions 9 and 10 are scored as 0, 2, or 4. Total scores range from 0 to 40; the cut-off point to identify hazardous alcohol intake is 8. A score between 16 and 19 indicates harmful alcohol use, and scores above 20 points indicate possible alcohol use disorder.

The Severity of Dependence Scale (SDS) (40) provides a self-reported measure of the psychological aspects of stimulants and alcohol dependence. A five-item, one-dimensional tool has a uniform scale for questions 1–4 from 0 (“never or almost never”) to 3 (“always”). Question 5 has the same scale with different signatures where 0 means “not difficult at all” and 3 means “impossible.” A score ranging from 0 to 15, where the cut-off score depends on the user’s drug type—a cut-off of ≤ 3 has been used for indexing alcohol dependence (41) and ≤ 5 for indexing amphetamine dependence (42).

The Drug Use Disorders Identification Test (DUDIT) measures self-reported problematic drug use (43). The DUDIT is an 11-item screening instrument, the first nine items are scored on a 5-point scale ranging from 0 to 4, and the last two are scored on a 3-point scale with values 0, 2, and 4. The overall score is calculated by summing the scores on all items, with a maximum score of 44. Previous research in adults established a score of > 24 for both sexes as a cut-off score for dependence (43).

Depression and anxiety are assessed with Hospital Anxiety and Depression Scale (HADS) (44), a 14 items two-dimensional tool. Each subscale consists of 7 items, scoring from 0 to 3. For each subscale, scores between 8 and 10 indicate mild depression/anxiety, and scores between 11 and 21 indicate depression/anxiety disorder.

Participants’ satisfaction with their life is assessed with The Satisfaction With Life Scale (SWLS) (45). SWLS is a short self-report instrument on which participants agree to five statements about life satisfaction on a seven-point Likert scale. A maximum score is 35, and higher scores indicate a high level of life satisfaction.

#### 2.8.3 Additional variables

The sensation-seeking trait is assessed with the Brief Sensation Seeking Scale (BSSS) (46)—an 8-items four-dimensional tool: (1) Disinhibition, (2) Boredom Susceptibility, (3) Thrill and Adventure Seeking, (4) Experience Seeking. Each subscale has two items rated on a scale from 1 (“strongly disagree”) to 5 (“strongly agree”). Results for the general score can range from 8 to 40, with higher scores indicating a higher sensation-seeking trait. Each subscale scores from 2 to 10, with a higher score indicating a higher sensation-seeking dimension rate.

Impulsivity is measured with the Short UPPS-P Impulsive Behavior Scale (SUPPS-P) (47), a 20-item four-dimensional tool: negative urgency (items 6, 8, 13, 15), lack of perseverance (items 1, 4, 7, 11), lack of premeditation (items 2, 5, 12, 19), sensation seeking (items 9, 14, 16, 18), and positive urgency (items 3, 10, 17, 20). Each item is scored from 1 (“strongly disagree”) to 4 (“strongly agree”). The minimum score on each subscale is 4, and the maximum is 16.

Participants’ coping with stress disposition is assessed with the Mini-COPE Stress Management Inventory, which consists of 28 statements divided into 14 strategies (2 statements in each strategy). In addition, strategies can be divided into three subscales: (1) problem-focused strategy, (2) emotion-focused strategy, and (3) avoidance strategy. Participants can answer on a 4-point scale from 1 (“I hardly ever do this”) to 3 (“I almost always do this”).

Emotion dysregulation is measured with a brief version of the Difficulties in Emotion Regulation Scale (DERS) (48). The tool consists of 18 items with six subscales: (1) Non-acceptance of emotional responses (items 7, 13, 14), (2) Difficulty engaging in goal-directed behavior (items 8, 12, 15), (3) Impulse control difficulties (items 9, 16, 18), (4) Lack of emotional awareness (items 1, 4, 6), (5) Limited access to emotion regulation strategies (items 10, 11, 17), and (6) Lack of emotional clarity (items 2, 3, 5). The scale has a five-point Likert scale from 1 (“Almost never”) to 5 (“Almost always”). Total scores range from 18 to 90 and 3 to 15 for each subscale. The higher result means more significant difficulties in emotion regulation.

### 2.9 Data analysis

We will use factorial design mixed-effects models to compare questionnaire battery scores between experimental groups and the control across measurements. In addition, we will perform an interrupted time series analysis to compare the longitudinal effects of the introduction of different types of interventions on longitudinal EMA outcomes.

We will include participants who complete at least 21 EMA assessments spread across the intervention testing period of 5 weeks. Furthermore, for the 6-month follow-up intervention effects retention analysis, we will include participants who complete at least three EMA assessments within the follow-up period. Moreover, in each intervention group, we will retain participants who log onto the app and use the long-term and short-term self-guided intervention modules at least four times and once, respectively, defined as minimal therapeutic exposure. Finally, for the secondary outcome analysis, we will include participants who complete the baseline and at least one follow-up assessment.

### 2.10 Data management

As described above, all data will be collected continuously throughout the duration of the study *via* the *Nałogometr* mobile app, available on App Store and Google Play, and stored on a secured server. Key project personnel, i.e., Principal Investigators (PIs) and Co-Investigators (CIs) will be the data steward and will be responsible for documenting and managing the data during the collection, analysis, and publication phases. Additional project personnel, i.e., project coordinators, data analysts, and data scientists, will receive the data as per instructions from the PIs and CIs in an anonymized format. Following publication processes, the data will be archived and stored on a similarly secured server.

Data documentation will include codebooks that document the following: data collection protocols, methodology, and sample; description of specific data sources, e.g., types of measures that correspond to each raw data unit.

As per the needs of the particular project phase and according to current research questions, data will be queried and exported as ASCII files and made available to the additional project staff. Such datasets will include an individual (anonymized) participant identifier code, demographic information, relevant variable labels, and values. According to the current research questions, additional project staff will perform any data transformations necessary for the final and published analyses. Any publications that result from the data collected will be prepared only with the use of anonymized (de-identified) datasets and will pertain only to aggregate-level results. Due to the expected absence of (high) risks for participants of this study, establishing a data monitoring committee is not necessary.

It will be the responsibility of each additional staff member to produce documentation of what and how were the data used for the research task they were involved in. This will include documentation on the decisions related to any data transformations and coding performed (including variable lists and definitions of the raw data used and how the derived variables were produced), as well as the analytical methods and techniques performed for any particular research task.

## 3 Discussion

This study protocol describes a randomized controlled trial designed to determine the effectiveness of self-guided psychological intervention modules delivered *via* a smartphone app (*Nałogometr*). We aim to examine whether and which interventions can effectively reduce craving and lapse risk in problematic alcohol or stimulant use. The embedded trial and analysis will evaluate the effectiveness of intervention modules for alcohol or/and stimulants use. In exploratory analyses, we investigate whether user engagement moderates or changes in psychological functioning measures (e.g., sensation seeking, impulsivity, stress coping, emotion regulation) mediate the effectiveness of the interventions. We hope to determine if these interventions delivered through mobile devices can be effective and contribute to future prevention or therapy addiction programs. The wide availability and accessibility of developed interventions are a substantial advantage. Mobile app interventions may reduce treatment barriers by staying anonymous and avoiding stigma and may be especially useful in places where professional help is unavailable (49–52). We plan a 6 months follow-up to conduct a longer-term evaluation and check the sustainability of the potential change in the user’s behavior–it is an essential factor for relapse prevention through the management of craving. Such a complex study of 13 conditions will allow us to understand better which long-term interventions are most effective. We include multiple intervention types to better assess their effectiveness across diverse individuals. One limitation of the present study could be that the participant dropout will be higher than expected based on previous research (53, 54). Due to the lack of personal contact with researchers and no additional incentives, e.g., prizes or monetary remuneration, the commitment to participate throughout the study may be lower, and motivation may decrease over time.

## Ethics statement

The studies involving human participants were reviewed and approved by the Institute of Psychology Polish Academy of Sciences Ethics Committee. Written informed consent for participation was not required for this study in accordance with the national legislation and the institutional requirements.

## Author contributions

MG, MS, KL, AB, and PM devised the initial plan for this study. KO, AB, PM, KL, and KSz did the first draft of the manuscript and prepared the final manuscript. KSo, KSz, MB, BW, and MN helped throughout the development of the intervention and gave valuable feedback to the present study protocol. All authors approved the final version of the manuscript submitted for publication.

## Footnotes

1
https://osf.io/kvqpa/?view_only=ccbbcb08c5c146cab37b0d2df23b585b

2
https://nalogometr.pl/
