# Corrigendum: Reducing craving and lapse risk in alcohol and stimulants dependence using mobile app involving ecological momentary assessment and self-guided psychological interventions: Protocol for a randomized controlled trial

**DOI:** 10.3389/fpsyt.2023.1146451

**Published:** 2023-02-01

**Authors:** Katarzyna Obarska, Alicja A. Binkowska, Przemysław Marcowski, Karol Szymczak, Karol Lewczuk, Katarzyna Sollich, Maria Banaszak, Bohdan Woronowicz, Małgorzata Nowicka, Maciej Skorko, Mateusz Gola

**Affiliations:** ^1^PredictWatch, Białystok, Poland; ^2^Institute of Psychology, Polish Academy of Sciences, Warsaw, Poland; ^3^DrugsTeam, NeuroCognitive Research Center, SWPS University of Social Sciences and Humanities, Warsaw, Poland; ^4^Institute of Psychology, The Maria Grzegorzewska University, Warsaw, Poland; ^5^Institute of Psychology, Cardinal Stefan Wyszynski University in Warsaw, Warsaw, Poland; ^6^Monar Association, Warsaw, Poland; ^7^Consulting Center Akmed, Warsaw, Poland

**Keywords:** mHealth, addiction, cognitive-behavioral therapy, mobile phone apps, EMA, substance use disorder (SUD), psychological intervention, mindfulness

In the published article, there was an error regarding the affiliations for Katarzyna Obarska, Maciej Skorko, and Mateusz Gola. As well as having affiliation 1, they should also have Institute of Psychology, Polish Academy of Sciences, Warsaw, Poland.

In the published article, there was an error regarding the affiliations for Alicja A. Binkowska. As well as having affiliation 1, she should also have DrugsTeam, NeuroCognitive Research Center, SWPS University of Social Sciences and Humanities, Warsaw, Poland.

In the published article, there was an error regarding the affiliations for Karol Szymczak. As well as having affiliation 1, he should also have Institute of Psychology, The Maria Grzegorzewska University, Warsaw, Poland.

In the published article, there was an error regarding the affiliations for Karol Lewczuk. As well as having affiliation 1, he should also have Institute of Psychology, Cardinal Stefan Wyszynski University in Warsaw, Warsaw, Poland.

In the published article, there was an error regarding the affiliations for Maria Banaszak. As well as having affiliation 1, she should also have Monar Association, Warsaw, Poland.

In the published article, there was an error regarding the affiliations for Bohdan Woronowicz. As well as having affiliation 1, he should also have Consulting Center Akmed, Warsaw, Poland.

The authors apologize for this error and state that this does not change the scientific conclusions of the article in any way. The original article has been updated.

