# Supplementary material for: Reducing craving and lapse risk in alcohol and stimulants dependence using mobile app involving ecological momentary assessment and self-guided psychological interventions: Protocol for a randomized controlled trial
Source: Front Psychiatry. 2022 Dec 14;13:1011585. doi: 10.3389/fpsyt.2022.1011585 (PMC9795071; doi:10.3389/fpsyt.2022.1011585)
Supplement: Supplementary file 1 [file Data_Sheet_1.docx]

**Table 1S.** Basic onboarding assessment.

| **Variable** | **Question** | **Answer** |
| --- | --- | --- |
| Gender |  | Female  Male  Other |
| Main  addiction |  | Substances:  Alcohol  Stimulants |
| Goal |  | abstinence  better self-control |
| Other addictions |  | I do not have  I have (select from the list)  Substances:  Alcohol  Heroin  Nicotine  Cannabis  Sedatives  Stimulants  Analgesics  Benzodiazepines  Behavior:  Pornography  Gaming  Gambling  Compulsive sex  Overeating  Other addiction?  (what?) |
| Age | Year of birth |  |
| Body Mass Index (BMI) | Height  Weight |  |
| Place of residence |  | less than 100,000 inhabitants  100,000 to 500,000 inhabitants  over 500,000 inhabitants |
| Dependence severity  (SDS for all addictions) | 1. Do behavior addiction was out of your control? 2. Was the prospect of stopping compulsive behavior causing anxiety? 3. Were you worried about your addictive behavior? 4. Would you like to quit your addictive behavior? 5. How difficult is it for you to stop and stay without addiction? | Answers:  0-Never / Almost never  1-Sometimes  2-Often  3-Always / Almost always  Answers:  0-No Difficulty  1-Difficult  2-Very difficult  Impossible |
| Addiction treatment | Have you ever been treated for your addiction? | in treatment now  in treatment in the past  never  Form of treatment:  Individual psychotherapy  Group psychotherapy  Pharmacotherapy  Psychiatric consultations  Self-help groups  Treatment in an inpatient clinic  Psychological assistance from a religious community  Other |
| Duration of abstinence | When did you last indulge in addictive behavior ? | 1-2 days  3-30 days  2-6 months  7 months and more |
| Family history of addictions | Does anyone in your immediate family suffer from addiction? | yes (select from the list)  no |

**Table 2S.** Further (non-obligatory) onboarding assessment

| **Variable** | **Question** | **Answer** |
| --- | --- | --- |
| Mental disorders | Have you ever been diagnosed by a psychiatrist for mental disorders? | No  Yes, which one?  Schizophrenia and other psychotic disorders  Mood disorders  Anxiety disorders  Personality disorders  Other (which?) |
| Frequency of addictive behavior |  | Less than once a month  Once a month  2-3 times a month  1-3 times a week  4-6 times a week  Daily |
| Age of addiction onset | How old were you when your problem with addiction began? |  |
| Number of attempts to stop the addiction | Considering the last 12 months, before installing our application, how many times have you tried to stop your addiction? |  |
| Financial costs of addiction | On average, how much money do you spend on your addiction per week? |  |
| Motivation to change | How much do you want to change your behavior/substance use? | scale 0-10  Not at all / Moderate / Very |

**Table 3S**. List of variables measured in daily ecological momentary assessment.

| **Variable** | **Question** | **Answer** |
| --- | --- | --- |
| Craving | How strong is your urge to use [substance] at the moment? | None/Incalculable (Scale 0-6) |
| Lapses* | Have you experienced a lapse since the last survey? | Yes  No |
| Mood | What is your mood at this moment? | Very negative / Very positive (Scale 0-10) |
| Arousal | How aroused are you feeling? | I'm calm / I'm nervous  (Scale 0-10) |
| Pressure | Do you feel any pressure at this point (that you have to do something or meet some of your or someone else's expectations)? | No pressure / Strong pressure (Scale 0-10) |
| Anxiety | How severe anxiety are you feeling at this moment? | No anxiety / Strong anxiety (Scale 0-10) |
| Procrastination | Are there any things you put off until later? | None / A lot (Scale 0-10) |
| Loneliness | Are you feeling lonely now? | Definitely not / Definitely yes (Scale 0-10) |
| Tiredness | How tired are you at this point? | Rested / Tired (Scale 0-10) |
| Anger | How much anger are you feeling right now? | No anger / Very angry  (Scale 0-10) |
| Hunger | Are you currently hungry? | I'm full / I'm hungry  (Scale 0-10) |
| Uncertainty | Is there anything currently causing a state of uncertainty in your life? | Yes  No  I do not know |

*Note.* If the user answers “yes” there are further questions about the number and date of lapses, negative consequences and risk factors. If the answer is “no” there is an additional question about protective factors.
